# Diazepam is not a direct allosteric modulator of α_1_‐adrenoceptors, but modulates receptor signaling by inhibiting phosphodiesterase‐4

**DOI:** 10.1002/prp2.455

**Published:** 2018-12-26

**Authors:** Lisa M. Williams, Xiaoji He, Tasneem M. Vaid, Alaa Abdul‐Ridha, Alice R. Whitehead, Paul R. Gooley, Ross A.D. Bathgate, Spencer J. Williams, Daniel J. Scott

**Affiliations:** ^1^ The Florey Institute of Neuroscience and Mental Health University of Melbourne Parkville Vic Australia; ^2^ School of Chemistry and Bio21 Molecular Science and Biotechnology Institute University of Melbourne Parkville Vic Australia; ^3^ Department of Biochemistry and Molecular Biology University of Melbourne Parkville Vic Australia; ^4^ The Bio21 Molecular Science and Biotechnology Institute University of Melbourne Parkville Vic Australia

**Keywords:** α_1_ adrenergic receptors, α_1_‐adrenoceptors, allosteric modulator, benzodiazepines, GPR68, phosphodiesterase, thermostabilized receptor

## Abstract

α_1A_‐ and α_1B_‐adrenoceptors (ARs) are G protein‐coupled receptors (GPCRs) that are activated by adrenaline and noradrenaline to modulate smooth muscle contraction in the periphery, and neuronal outputs in the central nervous system (CNS). α_1A_‐ and α_1B_‐AR are clinically targeted with antagonists for hypertension and benign prostatic hyperplasia and are emerging CNS targets for treating neurodegenerative diseases. The benzodiazepines midazolam, diazepam, and lorazepam are proposed to be positive allosteric modulators (PAMs) of α_1_‐ARs. Here, using thermostabilized, purified, α_1A_‐ and α_1B_‐ARs, we sought to identify the benzodiazepine binding site and modulatory mechanism to inform the design of selective PAMs. However, using a combination of biophysical approaches no evidence was found for direct binding of several benzodiazepines to purified, stabilized α_1A_‐ and α_1B_‐ARs. Similarly, in cell‐based assays expressing unmodified α_1A_‐ and α_1B_‐ARs, benzodiazepine treatment had no effect on fluorescent ligand binding, agonist‐stimulated Ca^2+^ release, or G protein activation. In contrast, several benzodiazepines positively modulated phenylephrine stimulation of a cAMP response element pathway by α_1A_‐ and α_1B_‐ARs; however, this was shown to be caused by off‐target inhibition of phosphodiesterases, known targets of diazepam. This study highlights how purified, stabilized GPCRs are useful for validating allosteric ligand binding and that care needs to be taken before assigning new targets to benzodiazepines.

**Abbreviations**
AC: adenylate cyclase
AR: adrenoceptor
CaM: calmodulin
CNS: central nervous system
CRE: cAMP response element
DDM: *n*‐dodecyl β‐D‐maltopyranoside
DMEM: Dulbecco's modified Eagle medium
GPCR: G protein‐coupled receptor
HEAT: [^125^I]‐2‐[[2‐(4‐hydroxy‐2‐iodophenyl)ethylamino]methyl]‐3,4‐dihydro‐2H‐naphthalen‐1‐one
HEK: human embryonic kidney
IP_3_: inositol 1,4,5‐trisphosphate
NMR: nuclear magnetic resonance
PAMs: positive allosteric modulators
PDE: phosphodiesterase
PKC: phosphokinase C
PLCβ: phospholipase (C‐β)
QAPB: quinazoline piperazine bodipy
SAR: structure–activity relationship
SD: standard deviation
STD: saturation transfer difference
TM: transmembrane
TOCSY: ^1^H total correlation spectroscopy
WT: wild‐type

## 1 INTRODUCTION

Adrenergic receptors, or adrenoceptors (ARs), are a family of G protein‐coupled receptors (GPCRs) that bind endogenous adrenaline and noradrenaline and are important for modulating the cardiovascular and nervous systems. GPCRs are seven α‐helical transmembrane (TM) proteins that bind ligands on the extracellular face shifting the conformational equilibria of the GPCR to active states and promoting cytoplasmic interactions with heterotrimeric G proteins and other signaling proteins.^1^There are three AR subfamilies, α_1_, α_2_, and β‐ARs, each comprising three receptor subtypes.^2^ The α_1_‐AR subtypes, α_1A_‐AR, α_1B_‐AR, and α_1D_‐AR couple to Gα_q/11_ G proteins to activate phospholipase C‐β (PLCβ) that catalyses the formation of the second messenger, inositol 1,4,5‐trisphosphate (IP_3_), thereby stimulating intracellular calcium mobilization. α_1_‐AR signaling stimulates smooth muscle contraction and thus α_1_‐AR antagonists are prescribed for hypertension and benign prostatic hyperplasia.^3^ α_1A_‐AR and α_1B_‐AR are also highly expressed in the brain, and transgenic rodent models have indicated α_1A_‐AR activation stimulates neurogenesis, while prolonged α_1B_‐AR stimulation promotes apoptotic neurodegeneration.^3^, ^4^ In the failing rodent heart, α_1A_‐AR stimulation drives adaptive hypertrophy, whereas chronic α_1B_‐AR activation causes maladaptive hypertrophy as a result of hemodynamic overload.^3^ Thus, selective α_1A_‐AR activation, or α_1B_‐AR blockade, could be useful therapeutic strategies for certain diseases.

While there are some α_1A_‐AR and α_1B_‐AR subtype‐selective ligands available, no highly α_1_‐AR subtype‐selective drugs have been approved for use in the clinic. The highly similar ligand binding sites in closely related AR subtypes makes identifying subtype‐selective ligands challenging. Allosteric ligands, on the other hand, bind to distinct sites in the receptor and can modulate the activity of the receptor in response to agonist binding. As allosteric sites are less conserved between receptor subtypes it may be possible that allosteric modulators offer scope for achieving subtype selectivity. Several ligands have been reported to act as allosteric modulators of α_1A_‐AR and α_1B_‐AR, including the conotoxin ρ‐TIA,^5^ 9‐aminoacridine,^6^ and the benzodiazepines diazepam, lorazepam, and midazolam.^7^ However, the exact structural mechanisms by which these ligands modulate the receptors are unknown.

Benzodiazepines are positive allosteric modulators (PAMs) of GABA_A_R ion channels and are widely prescribed as sedatives, anxiolytics, anticonvulsants, and myorelaxants.^8^ The benzodiazepines, diazepam and lorazepam, inhibit Ca^2+^ oscillations in pulmonary artery smooth muscle cells, suggestive of off‐target interactions with ARs.^9^ Waugh et al. 7 postulated that benzodiazepines directly bind to α_1_‐ARs. Diazepam, lorazepam, and midazolam also directly competed with the α_1_‐AR antagonist [^125^I]‐2‐[[2‐(4‐hydroxy‐2‐iodophenyl)ethylamino]methyl]‐3,4‐dihydro‐2H‐naphthalen‐1‐one (HEAT) on α_1A/B/D_‐AR expressing COS‐1 cells.^7^ Functional IP_3_ assays demonstrated potentiation of agonist responses at α_1A/B/D_‐AR‐expressing cells by these benzodiazepines, suggestive of allosteric interaction. However, no further studies have been reported on the mechanism of benzodiazepine modulation of α_1_‐ARs, or the receptor binding site. We recently engineered thermostabilized mutants of α_1A_‐AR and α_1B_‐AR, which enabled binding epitope determination of orthosteric ligands with nuclear magnetic resonance (NMR) spectroscopy.^10^ In this work we sought to use these thermostabilized receptors and our NMR approach to further understand how benzodiazepines bind to and modulate α_1_‐ARs so as to apply this information as a starting point for developing more selective modulators targeting this allosteric site. Instead we observed no evidence for the direct binding of several benzodiazepines to purified α_1A_‐AR and α_1B_‐AR using NMR and fluorescent ligand binding assays. Cell‐based binding and calcium signaling assays with wild‐type (WT) α_1_‐ARs also failed to detect any direct modulatory effects of diazepam on these receptors. While diazepam could positively modulate the stimulation of a cAMP response element (CRE) reporter through α_1_‐AR activation, this was found to be driven through the ability of diazepam to inhibit phosphodiesterases (PDEs), a known target of some benzodiazepines. This study highlights how purified GPCRs can be used to directly investigate allosteric modulator mechanisms that have been proposed from cell‐based assays, where off‐target actions are difficult to control for.

## 2 MATERIALS AND METHODS

### 2.1 Benzodiazepine preparation

Nordiazepam and diazepam were synthesized from 2‐amino‐5‐chlorobenzophenone using the method of Sternbarch et al.^11^ *N*‐hydroxyethyl‐nordiazepam was synthesized from nordiazepam according to the method of Archer et al.^12^ Further details of benzodiazepine preparation and chemical synthesis can be found in Supporting Information.

### 2.2 Protein expression, purification, and binding assays

α_1A_‐AR variant A4 and α_1B_‐AR variant #15 were expressed in *Escherichia coli* and purified as described previously.^10^ Stabilized rat neurotensin receptor 1 (enNTS_1_) was expressed and purified as described by Bumbak et al.^13^ BODIPY‐FL‐prazosin (QAPB [quinazoline piperazine bodipy]) competition binding assays were performed as described previously.^10^ Briefly, 2 nmol of purified receptor, with C‐terminal mCherry‐Avi tag fusion, were incubated with 100 μL Dynabeads^®^ MyOne Streptavidin T1 paramagnetic beads (Life Technologies, Carlsbad, CA, USA) in 10 mL binding buffer (1× PBS, 0.05% *n*‐dodecyl β‐D‐maltopyranoside [DDM], 10 mmol/L EDTA), at 4°C for 1.5 hours. The receptor‐coated beads were washed in binding buffer, and then aliquoted into a KingFisher 96‐DeepWell™plate (Thermo Fisher Scientific, Waltham, MA, USA) at approximately 20 pmol of receptor per well. A Kingfisher 96 magnetic particle processor was then used to transfer the beads into plates containing QAPB and various competitors, which was incubated for 2 hours at 22°C with gentle mixing. The beads were then washed for 1 minute in binding buffer then transferred to 100 μL binding buffer in black Greiner nonbinding 96‐well plates. QAPB fluorescence was measured using 485/12 nm excitation and 520/10 nm emission filters, while mCherry fluorescence was measured using 544 nm excitation and 590/10 nm emission filters in an Omega POLARstar plate reader (BMG Labtech, Ortenberg, Germany).

### 2.3 NMR spectroscopy

Stock solutions of 100 mmol/L diazepam and lorazepam were prepared in deuterated DMSO. Stock solutions of 5 mmol/L prazosin were prepared in deuterated methanol. Saturation transfer difference (STD) NMR samples constituted 5 μmol/L α_1A_‐AR A4, or 5 μmol/L enNTS1, and 500 μmol/L test ligands with and without competition of 10 μmol/L prazosin in 500 μL of DDM buffer (0.05% [or 1 mmol/L] DDM, 50 mmol/L potassium phosphate, 100 mmol/L NaCl, 10% ^2^H_2_O, pH 7.4) in 5 mm NMR tubes. STD NMR was performed as described previously on a 700 MHz Bruker Avance IIIHD spectrometer using a cryogenically cooled triple resonance probe^10^ and the resultant spectra were analyzed using Mnova NMR 10 (Mestrelab, Santiago de Compostela, Spain).

Diazepam and lorazepam assignments were confirmed with two‐dimensional ^1^H total correlation spectroscopy (TOCSY) and ^1^H correlated spectroscopy spectra acquired under the same solution conditions as the STD NMR experiments and in the presence of receptor. Spectra were typically acquired at 25°C with 10 ppm spectral widths and 2K data points in the direct dimension and 256 data points in the indirect dimension. TOCSY spectra were run with a spin lock time of 60 ms.

### 2.4 Whole cell QAPB binding and Ca^2+^ mobilization assays

Saturation binding of QAPB in the absence or presence of 50 μmol/L diazepam was measured using COS‐7 cells stably expressing WT human α_1A_‐AR and α_1B_‐AR as previously described.^10^ Cells were gently suspended with pipetting into 1.4 mL of phenol red‐free Dulbecco's modified Eagle medium (DMEM) at 20°C, resulting in a concentration of approximately 6 000 00 cells/mL. Fifty microliter of the cell suspension was added to each well of a V‐bottom 96‐well plate (Sarstead, Nümbrecht, Germany). A further 50 μL of phenol red‐free DMEM containing QAPB at varying concentrations was added to make final QAPB concentrations of 0, 0.78, 1.56, 3.12, 6.25, 12.5, 25, and 50 nmol/L. Separate solutions of these QAPB concentrations were made in the presence of 50 μmol/L diazepam. To determine nonspecific binding, cells were exposed to QAPB at the same concentrations as above, but in the presence of 100 μmol/L phentolamine. The cells were incubated with ligands for 1 hour at 20°C prior to detection of bound QAPB with flow cytometry using CytoFLEX LX flow cytometer (Beckman Coulter, Brea, CA, USA).

Intracellular Ca^2+^ mobilization assays were performed on nontransfected COS‐7 cells and cells stably expressing either α_1A_‐AR or α_1B_‐AR. Cells were seeded at 25 000 cells per well into 96‐well culture plates and allowed to grow overnight at 37°C, 5% CO_2_. Cells were washed twice with Ca^2+^ assay buffer (150 mmol/L NaCl, 2.6 mmol/L KCl, 1.2 mmol/L MgCl_2_, 10 mmol/L D‐glucose, 10 mmol/L HEPES, 2.2 mmol/L CaCl_2_, 0.5% [w/v] BSA, and 4 mmol/L probenecid, pH 7.4) and then incubated in Ca^2+^ assay buffer containing 1 mmol/L Fluo‐4‐AM for 1 hour in the dark at 37°C and 5% CO_2_. After two washes with Ca^2+^ assay buffer and the addition of phenylephrine solutions (or co‐addition of phenylephrine and benzodiazepines) fluorescence was measured for 1.5 minute in a Flexstation plate reader (Perkin Elmer, Waltham, MA, USA) using an excitation wavelength of 485 nm and emission wavelength of 520 nm.

### 2.5 CRE reporter assays

cAMP response element (CRE) response assays were performed as previously described by transfecting WT α_1A_‐ and α_1B_‐AR stably expressing COS‐7 cells, parental COS‐7 cells or parental HEK293T cells, with a β‐galactosidase expression plasmid under the control of the CRE promoter.^10^ CRE reporter‐transfected cells were seeded into 96‐well culture plates for 24 hours before treatment with various compounds for 6 hours at 37°C 5% CO_2_. Media was aspirated, and the cells frozen at −80°C for at least 24 hours before measurement of cellular β‐galactosidase expression using chlorophenol red‐β‐D‐galactopyranoside. Ligands were made up in 0.5% FBS DMEM media. Benidipine (10 μmol/L), bicuculline (30 μmol/L), IBMX (500 μmol/L), or rolipram (10 μmol/L) were co‐added with 0.1 μmol/L phenylephrine, or 10 μmol/L forskolin with and without diazepam or lorazepam (50 μmol/L). Inhibitors W‐7 hydrochloride (10 μmol/L), 2‐APB (40 μmol/L), (R)‐(+)‐Bay 8644 (4 μmol/L), and KN‐93 (20 μmol/L) were preincubated for 30 minute prior to addition of 50 μmol/L diazepam, and 10 μmol/L forskolin or 0.1 μmol/L phenylephrine for 6 hours. HEK293T cells transiently transfected with GPR68 and pCRE were stimulated with sodium bicarbonate‐free, low glucose DMEM, supplemented with 20 mmol/L HEPES and 0.5% FBS at pH 6.8, 7.0, 7.2, 7.4, and 7.8. The pH was adjusted at 37°C with NaOH. Cells were incubated for 6 hours at 37°C, in a room atmosphere incubator. Diazepam or lorazpam were made up in media at pH 7.2 or 7.8, co‐added with rolipram and stimulated for 6 hours in above conditions.

### 2.6 Data analysis

Nuclear magnetic resonance (NMR) data were processed in Topspin 3.5 using squared cosine‐bells in both dimensions and zero‐filled once, prior to Fourier‐transformation. All other data was analyzed with Graphpad prism 7 (San Diego, CA, USA). All error bars are standard deviation (SD) from three independent experiments. Competition binding assays were performed in duplicate wells. Curves were fit with one site nonlinear regression. For whole cell QAPB binding, the mean QAPB fluorescence intensity (MFI) of at lease 5000 cells was measured, and plotted against QAPB concentration. Values reported represent the mean and SD of at least three *K*_d_ values calculated from separate measurements. Ca^2+^ mobilization data were performed in triplicate wells and normalized to the peak response elicited by 3 μmol/L ionomycin. CRE assay was conducted in triplicate, and data were normalized to the response elicited by the vehicle. Curves were fitted with three variable nonlinear regressions. For GPR68 assays, responses were normalized to CRE response at pH 6.8 (100%) and pH 7.8 (0%).

## 3 RESULTS

**Figure 1:**
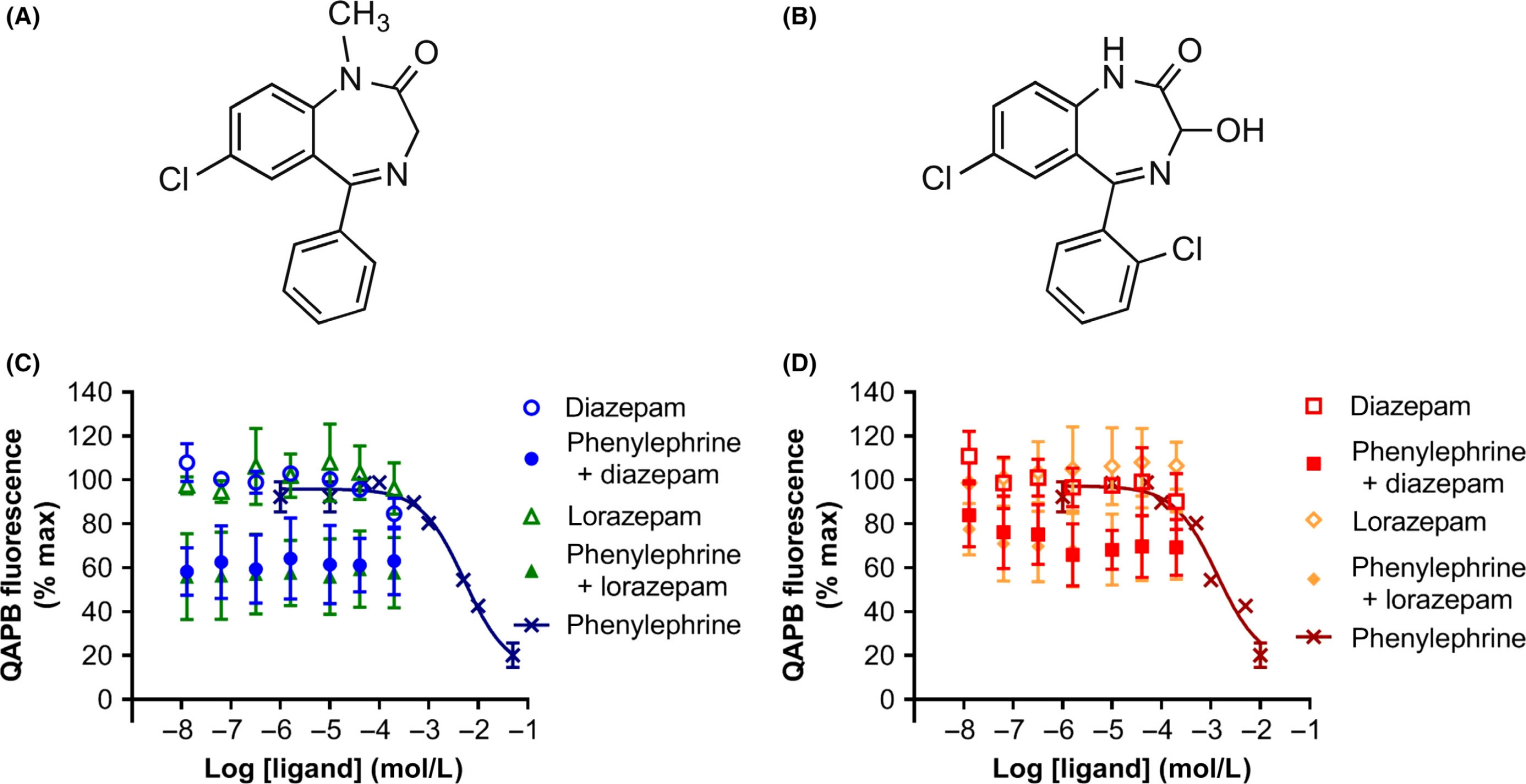
Diazepam and lorazepam quinazoline piperazine bodipy (QAPB) competition binding assays. Chemical structures of (A) diazepam and (B) lorazepam. QAPB competition binding at (C) α_**1A**_‐AR A4 and (D) α_1B_‐AR #15. Binding was normalized with 100% representing 10 nmol/L QAPB without competition and 0% 10 nmol/L QAPB with competition with 10 μmol/L prazosin. Competition was performed with increasing concentrations of phenylephrine, diazepam, and lorazepam, or diazepam and lorazepam in the presence of 2.5 mmol/L phenylephrine. Data are mean ± SD of three independent experiments performed in duplicate

Previously the benzodiazepines lorazepam,diazepam, and midazolam were shown to behave as positive modulators of α_1_‐AR agonists in cell lines overexpressing α_1_‐ARs, with low micromolar potencies.^7^ However, direct binding of benzodiazepines to purified α_1_‐AR proteins has never been demonstrated. Thermostabilized GPCRs are receptors containing mutations that improve the protein stability upon solubilization and purification using detergents.^14^ The retention of natural receptor pharmacology enables thermostabilized receptors to be used for structural biology^15^ and for probing the mechanisms and kinetics of ligand binding in a purified system.^10^, ^13^, ^16^ The thermostabilized variants α_1A_‐AR A4 and α_1B_‐AR #15 were recently described and exhibit the stability required to probe the binding of benzodiazepines to the purified receptors.^10^ We hypothesized that if benzodiazepines are direct allosteric modulators of α_1_‐AR, then they should influence the binding of orthosteric antagonists and/or agonists to α_1_‐AR. Biotinylated α_1A_‐AR A4 or α_1B_‐AR #15 was immobilized onto streptavidin‐coated paramagnetic beads and placed in a 10 nmol/L solution of fluorescent‐labeled BODIPY‐FL prazosin (QAPB), an approximately *K*_d_ concentration, with serial dilutions of validated competitors,diazepam or lorazepam, for 2 hours. The *K*_d_ of QAPB at α_1A_‐AR A4 and α_1B_‐AR #15 has been previously reported to be 11.6 and 8.5 nmol/L respectively.^10^ The agonist phenylephrine displaced QAPB at both receptor subtypes in an expected dose‐dependent manner (Figure 1A,B). Conversely, neither diazepam nor lorazepam displaced QAPB at either receptor, even at concentrations of up to 200 μmol/L (Figure 1A,B). As diazepam is predicted to bind to an allosteric site distinct from the orthosteric site, where QAPB binds, this discrepancy could be due to noncompetitive binding of the two ligands. However, the ability of diazepam to increase the potency and efficacy of the α_1_‐AR agonist phenylephrine suggests that allosteric binding of diazepam influences the affinity of phenylephrine for the receptors. Thus, QAPB competition binding assays were performed at α_1A_‐AR A4 and α_1B_‐AR #15 where a sub‐IC_50_ concentration of phenylephrine (2.5 mmol/L) was included and the ability of increasing concentrations of diazepam to positively modulate QAPB displacement by phenylephrine was monitored. No cooperativity between diazepam and phenylephrine was observed at either receptor subtype. These data suggest that diazepam is either not binding to the thermostabilized α_1_‐ARs, has a very low affinity interaction, or is allosterically binding, but not able to modulate phenylephrine binding in this purified system.

**Figure 2:**
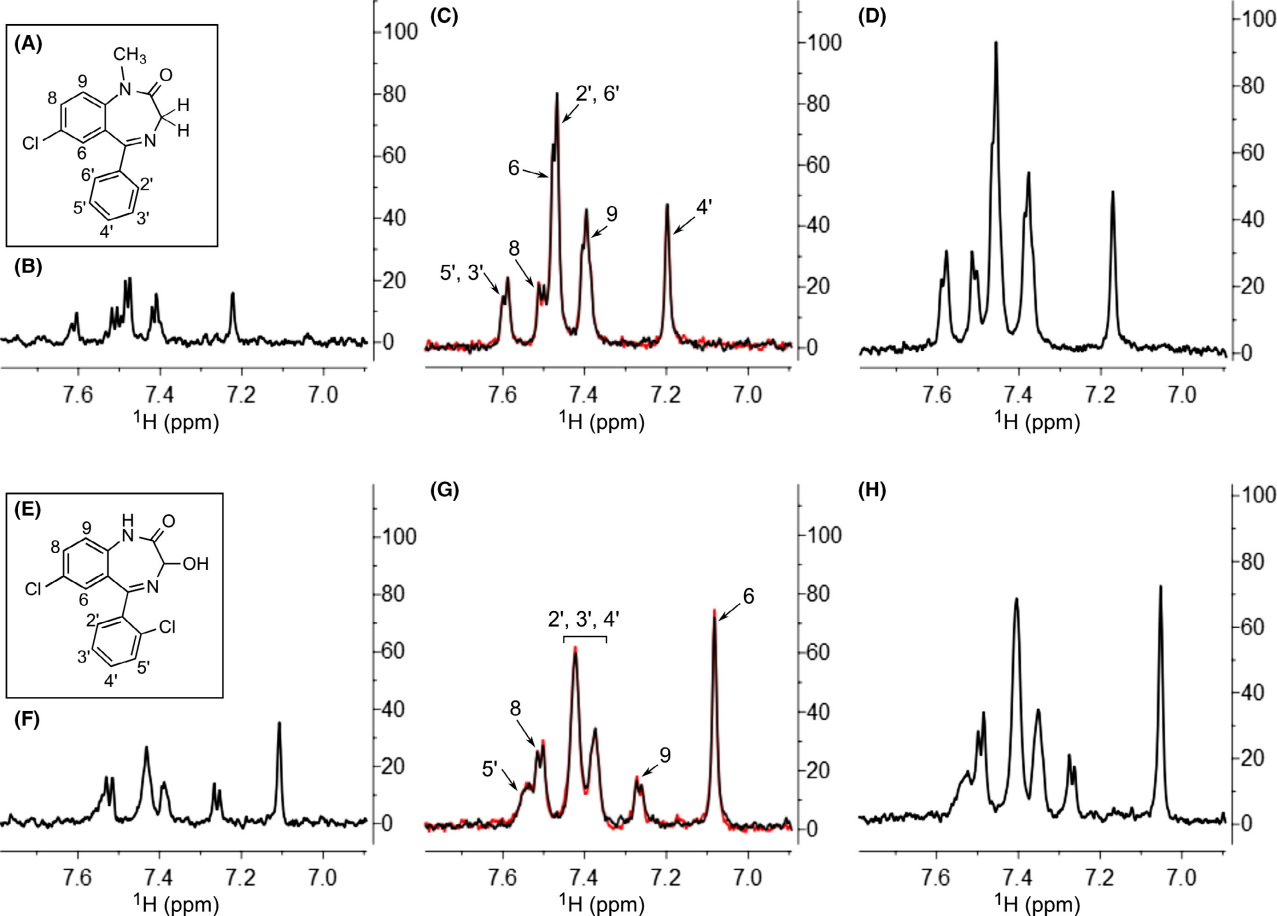
Saturation transfer difference (STD) nuclear magnetic resonance (NMR) of diazepam and lorazepam. (A) Structure of diazepam with nuclei observed in the subsequent spectra labeled. (B) STD NMR spectrum of the aromatic protons of diazepam incubated with *n*‐dodecyl β‐D‐maltopyranoside (DDM). (C) STD NMR spectrum of diazepam incubated with α_1A_‐AR A4 solubilized in DDM without (black spectrum) and with prazosin (red spectrum). Resonances are labeled as in (A). (D) STD NMR spectrum of diazepam incubated with enNTS_1_ solubilized in DDM. (E) Structure of lorazepam with nuclei observed in the subsequent spectra labeled. (F) STD NMR spectrum of the aromatic protons of lorazepam incubated with DDM. (G) STD NMR spectrum of lorazepam incubated with α_1A_‐AR A4 solubilized in DDM without (black spectrum) and with prazosin (red spectrum). Resonances are labeled as in (E). (H) STD NMR spectrum of lorazepam incubated with enNTS_1_ solubilized in DDM

Saturation transfer difference (STD) NMR is a ligand‐observed experiment that is especially sensitive at monitoring ligands that bind weakly to proteins (*K*_d_ > 1 μmol/L) and does not require labeled ligands or proteins.^17^ We recently applied STD NMR to the study of orthosteric agonist binding at α_1A_‐AR A4 and α_1B_‐AR #15.^10^ Here, STD NMR was applied to determine if diazepam and lorazepam bind to purified α_1A_‐AR A4. STD NMR signals were observed for diazepam and lorazepam (Figure 2A,E) when incubated with DDM micelles that were not loaded with protein (Figure 2B,F), suggesting significant nonspecific interactions with detergent. Stronger STD NMR signals were observed for diazepam and lorazepam when incubated with purified α_1A_‐AR A4, solubilized in DDM (Figure 2C,G) and this signal was not reduced upon the addition of 10 μmol/L prazosin as a competitor, suggesting either the detection of nonspecific or allosteric binding of the benzodiazepines to α_1A_‐AR A4. However, strong STD NMR signals were also observed when diazepam and lorazepam were studied in the presence of an unrelated receptor, stabilized neurotensin receptor 1 (enNTS_1_)^13^ (Figure 2D,H), suggesting that the STD NMR signals of diazepam and lorazepam in all these experiments were due to nonspecific interactions with detergent micelles.

**Figure 3:**
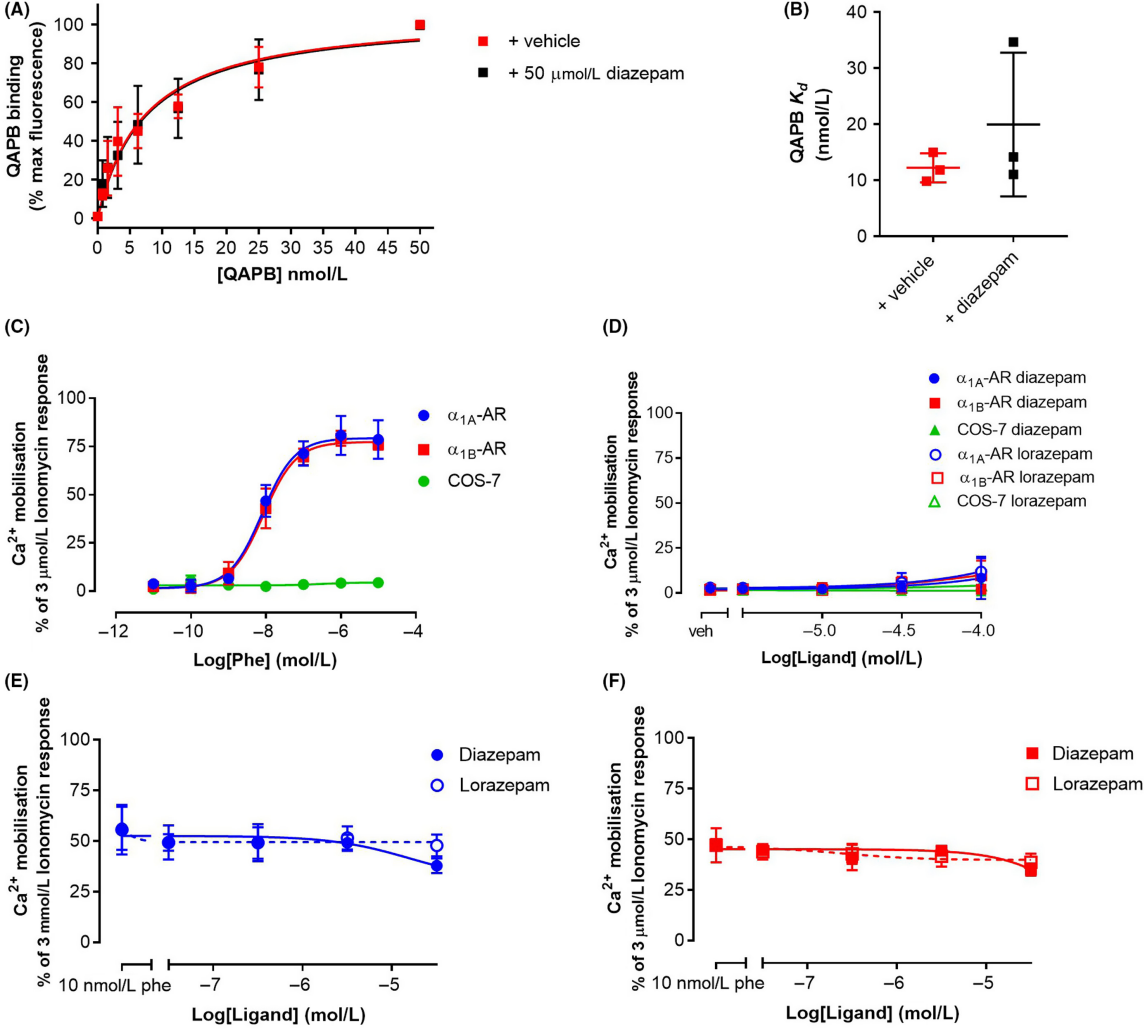
Cell‐based assays of benzodiazepine action at WT α_1_‐ARs. (A) Pooled quinazoline piperazine bodipy (QAPB) saturation binding curves in the absence (+ vehicle) and presence of 50 μmol/L diazepam and (B) the resultant *K*_d_ values from each experimental replicate. (C) Phenylephrine‐induced intracellular Ca^2+^mobilization in COS‐7 cells stably expressing either WT α_1A_‐AR (blue circles), WT α_1B_‐AR (red squares) or no receptor (green circles). (D) Diazepam and lorazepam did not stimulate Ca^2+^ mobilization in any of the cell lines, nor did they potentiate phenylephrine stimulation of Ca^2+^ mobilization in (E) WT α_1A_‐AR stable cells or (F) WT α_1B_‐AR stably expressing cells

Having failed to detect any evidence for specific interactions between diazepam and lorazepam and α_1A_‐AR A4 and α_1B_‐AR #15, we sought to validate the work of Waugh et al.^7^ by measuring competitive binding and positive agonist modulation of benzodiazepines using cells overexpressing WT human α_1A_‐AR and α_1B_‐AR. A benzodiazepine related to diazepam and midazolam, was previously shown to compete with iodinated HEAT, a selective α_1_‐AR antagonist, at cells expressing α_1A_‐AR, α_1B_‐AR or α_1D_‐AR.^7^ HEAT is thought to bind in the orthosteric binding site, thus we sought to validate this competitive binding behavior of benzodiazepines using QAPB on COS‐7 cells stably expressing unmodified α_1B_‐AR. Flow cytometry‐based QAPB saturation binding assays were performed in the absence or presence of 50 μmol/L diazepam. QAPB exhibited the expected affinity for WT α_1B_‐AR (*K*_d_ = 12 ± 3 nmol/L), which was not significantly different in the presence of 50 μmol/L diazepam (*K*_d_ = 20 ± 13 nmol/L) (Figure 3A,B).

**Figure 4:**
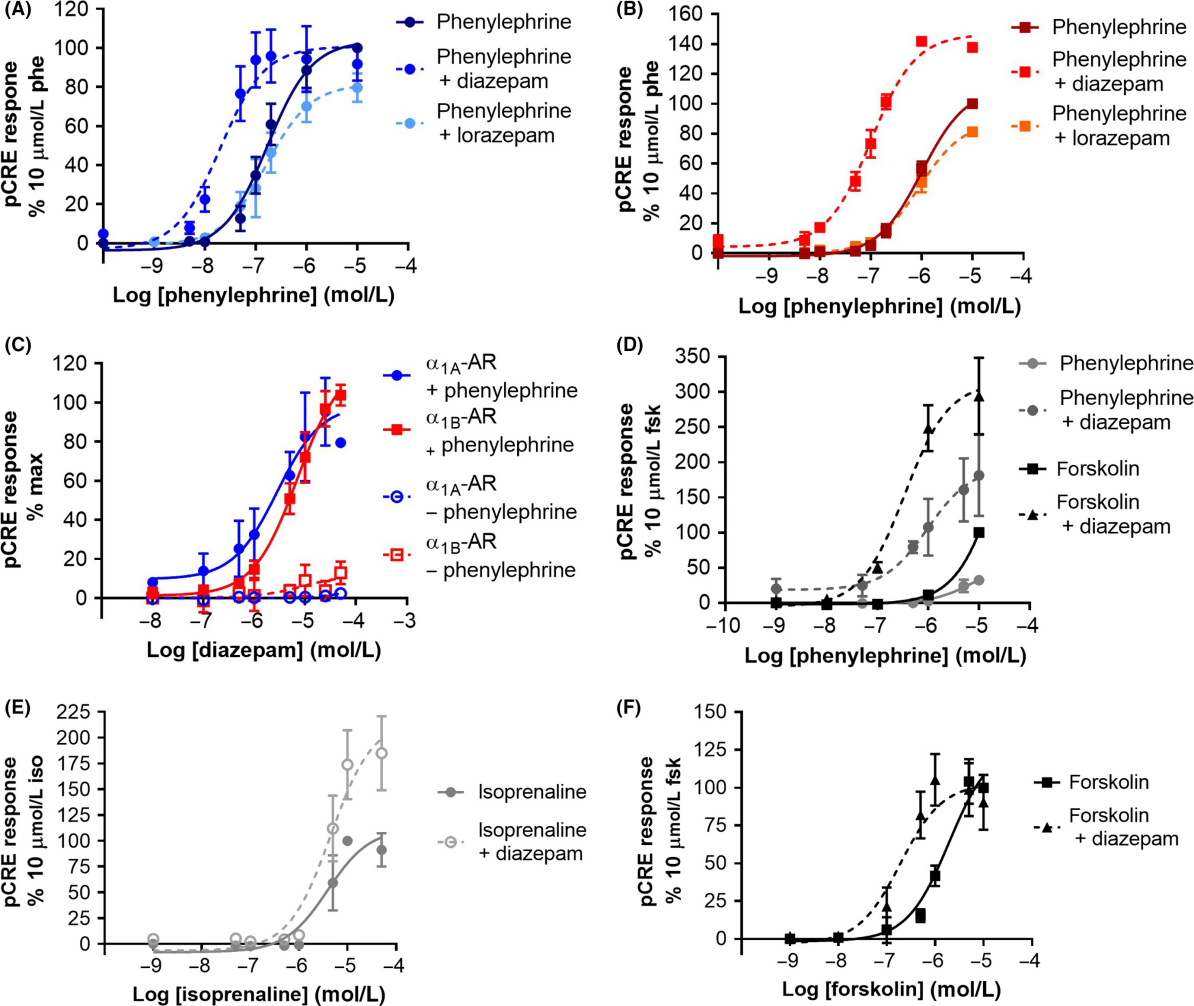
Modulation cAMP response element (CRE) activation by diazepam. Phenylephrine‐induced CRE response in the absence, or presence of 50 μmol/L diazepam or lorazepam at (A) COS‐7 cells stably expressing wild‐type (WT) α_1A_‐AR or (B) COS‐7 cells stably expressing WT α_1B_‐AR. (C) Dose–response curves of diazepam modulating the phenylephrine‐induced CRE response in WT α_1A_‐AR (200 nmol/L phenylephrine used) and WT α_1B_‐AR (800 nmol/L phenylephrine used) expressing COS‐7 cells. (D) Phenylephrine‐ and forskolin‐induced CRE response in the absence, or presence of 50 μmol/L diazepam at untransfected COS‐7 cells. (E) Isoprenaline‐induced CRE response in the absence, or presence of 50 μmol/L diazepam at untransfected human embryonic kidney (HEK) 293T cells. (F) Forskolin‐induced CRE response in the absence, or presence of 50 μmol/L diazepam at untransfected HEK293T cells

To probe whether allosteric binding of diazepam could modulate α_1_‐AR signaling, COS‐7 cells stably expressing human α_1A_‐AR and α_1B_‐AR were used for calcium mobilization assays. The agonist, phenylephrine, stimulated calcium mobilization in α_1_‐AR expressing cells, but not untransfected COS‐7 cells, with expected potencies (Figure 3C). To probe positive modulation of this response, α_1A_‐AR and α_1B_‐AR expressing cells were treated with an EC_50_ concentration of phenylephrine (10 nmol/L) and increasing concentrations of diazepam or lorazepam before measuring the mobilization of intracellular calcium 5 minute after treatment. Treatment with these benzodiazepines (0 to 50 μmol/L), had no effect in the absence (Figure 3D), or presence of phenylephrine on both receptor expressing cell lines (Figure 3E,F). To gain a more complete measure of phenylephrine‐induced α_1_‐AR signaling over a 6‐h stimulation period, a CRE‐reporter gene assay was employed. In this assay, diazepam positively modulated the potency of phenylephrine at α_1A_‐AR and α_1B_‐AR expressing cells and also increased the *E*_max_ of phenylephrine at α_1B_‐AR expressing cells (Figure 4A,B and Table 1). Diazepam modulated the phenylephrine CRE response with potencies of 6.5 ± 4.8 μmol/L on α_1A_‐AR and 7.8 ± 1.9 μmol/L on α_1B_‐AR (mean EC_50_ ± SD from three experiments, Figure 4C). Critically, diazepam treatment in the absence of phenylephrine did not induce a CRE response (Figure 4C). However, diazepam positively modulated the phenylephrine‐induced CRE response on COS‐7 cells that do not express α_1A_‐AR and α_1B_‐AR (Figure 4D), suggesting that the action of diazepam on CRE signaling is independent of α_1_‐AR stimulation.

**Table 1:** Structure activity relationship governing benzodiazepine modulation of the CRE response 
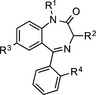

Name	R1=	R2=	R3=	R4=	phe pEC_50_	phe *E*_max_ (% veh)	fsk *E*_max_ (% veh)
DMSO	N.A.	N.A.	N.A.	N.A.	6.07 ± 0.07	100	100
nordiaz	H	H_2_	Cl	H	6.27 ± 0.11	114 ± 3	107 ± 11
diaz	CH_3_	H_2_	Cl	H	6.99 ± 0.08^a^	146 ± 3^a^	167 ± 3^a^
loraz	H	OH	Cl	Cl	6.05 ± 0.12	89 ± 5	112 ± 16
temaz	CH_3_	OH	Cl	H	6.49 ± 0.1^a^	127 ± 2^a^	N.D.
oxaz	H	OH	Cl	H	6.44 ± 0.10^a^	121 ± 8^a^	N.D.
OH‐diaz	C_2_H_4_OH	H_2_	Cl	H	6.37 ± 0.09^a^	115 ± 9	N.D.
7‐Br	CH_3_	H_2_	Br	H	7.10 ± 0.08^a^	172 ± 11^a^	146 ± 31
7‐Ph	CH_3_	H_2_	Ph	H	6.56 ± 0.06^a^	118 ± 6^a^	138 ± 16

The potency of each benzodiazepine at modulating the phenylephrine‐stimulated cAMP response element (CRE) response at α_1B_‐AR expressing COS‐7 cells (phe pEC_50_) are expressed as the mean ± SD from three independent experiments. The maximum CRE response induced by either phenylephrine stimulation of α_1B_‐AR expressing COS‐7 cells (phe *E*_max_), or forskolin at untransfected human embryonic kidney 293T cells (fsk *E*_max_), in the presence of each benzodiazepine are expressed as percentages compared to the vehicle (DMSO) from three independent experiments.

nordiaz, nordiazepam; diaz, diazepam; loraz, lorazepam; temaz, temazepam; oxaz, oxazepam; OH‐nor, *N*‐hydroxyethyl‐nordiazepam; 7‐Br, 7‐Bromo‐diazepam; 7‐Ph, 7‐phenyl‐diazepam; N.D., indicates not determined.

a Statistically significant difference (*P* < 0.05), evaluated using one‐way ANOVA and Tukey multiple comparisons tests.

All α_1_‐ARs signal primarily through Gα_q/11_ G proteins, which activate the effector protein phospholipase C (PLC). PLC catalyses the formation of diacylglycerol (DAG), which then activates phosphokinase C (PKC), and IP_3_ causing Ca^2+^ release from the endoplasmic reticulum via InsP3R Ca^2+^ channels.^18^ α_1_‐AR activation also has a secondary effect of stimulating cAMP production via calmodulin (CaM)‐stimulated adenylate cyclase (AC).^19^ PKC and secondary G protein coupling may also play a role in AC activation.^20^, ^21^ CaM family kinases I and IV are activated by CaM which phosphorylate the transcription factor cAMP response binding protein, leading to its activation, and upregulation of CRE genes.^22^ Thus, there are many molecular targets in the CRE pathway that benzodiazepines may be interacting with to positively modulate the α_1_‐AR CRE response. To assess the specificity of this action of diazepam, CRE assays were performed on human embryonic kidney (HEK) 293‐T cells, which endogenously express β‐ARs, treated with the β_2_‐AR agonist isoprenaline. Treatment with diazepam at 50 μmol/L significantly increased the CRE efficacy of isoprenaline at HEK293‐T cells (Figure 4E), suggesting that diazepam modulation of the CRE response is not specific to α_1_‐AR stimulation. Furthermore, 50 μmol/L diazepam positively modulated the potency of the AC activator forskolin in HEK293‐T cells (Figure 4F), indicating that diazepam is not modulating CRE activity at the receptor level, but at some other step of the signaling pathway.

**Figure 5:**
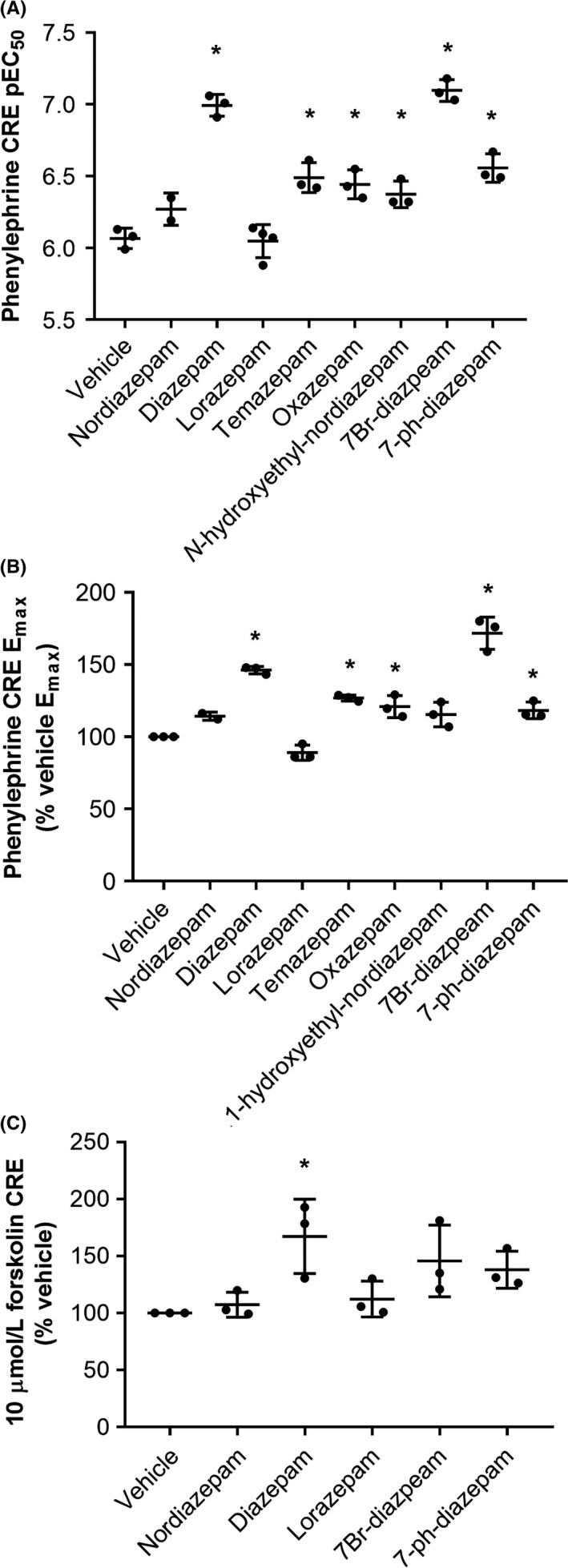
Structure–activity relationship (SAR) of benzodiazepine modulation of the cAMP response element (CRE) response. (A) Replicate phenylephrine‐induced CRE pEC_50_ values, and (B) maximum phenylephrine‐induced CRE responses, in the presence of 50 μmol/L of each indicated benzodiazepine at wild‐type α_1B_‐AR stably expressing COS‐7 cells. (C) Replicate forskolin (10 μmol/L)‐induced CRE responses in the presence of 50 μmol/L of each indicated benzodiazepine at human embryonic kidney 293‐T cells. The means are indicated with horizontal lines, the error bars represent SD values, and * indicates significant statistical difference to vehicle, measures with one‐way ANOVA and Tukey multiple comparisons test

The structure–activity relationship (SAR) of benzodiazepines at their clinical target, GABA_A_R is well understood, and thus the SAR of CRE modulation was investigated to gain insight into what the underlying molecular target may be. The benzodiazepines tested, and their modifications compared to diazepam, are listed in Table 1. Interestingly, the *N*‐methyl group of diazepam, which is lacking in nordiazepam, was found to be critical for positive modulation of the phenylephrine‐induced CRE activation in α_1B_‐AR expressing COS‐7 cells and forskolin stimulation of CRE in untransfected HEK cells (Figure 5 and Table 1). Furthermore, the GABA_A_R‐inactive benzodiazepine, 7‐phenyl‐diazepam, was able to modulate the phenylephrine‐induced CRE activation, although its actions on forskolin failed to reach statistical significance (Figure 5 and Table 1). This defined SAR indicated that the benzodiazepine mechanism driving this positive modulation of phenylephrine was likely being driven by a specific binding interaction with a target other than GABA_A_R in the cells rather than nonspecific interactions, for example, with the cell membrane.

**Figure 6:**
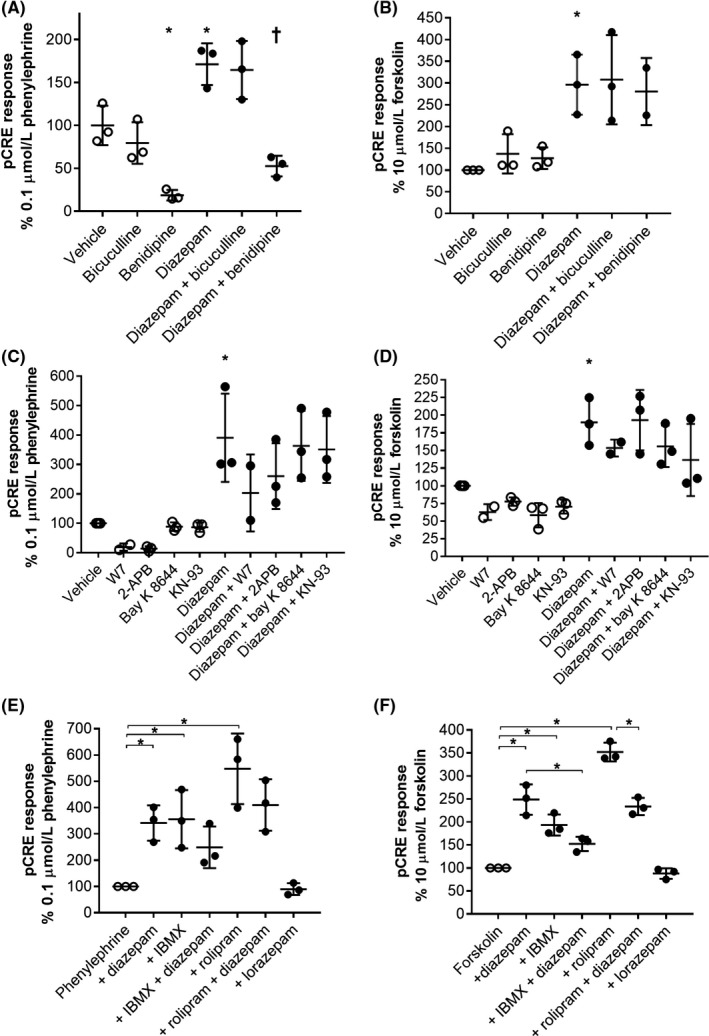
Screening potential benzodiazepine targets with inhibitors in wild‐type α_1A_‐AR stably expressing COS‐7 cells. (A) 0.1 μmol/L phenylephrine‐induced cAMP response element (CRE) response, or (B) 10 μmol/L forskolin‐induced CRE response in the absence (open circles) or presence (solid circles) of diazepam and GABA_A_R antagonist bicuculline or Ca^2+^ channel inhibitor benidipine. * indicates statistically significant difference from vehicle and † significantly different from phenylephrine + diazepam treatment. (C) 0.1 μmol/L phenylephrine‐induced CRE response, or (D) 10 μmol/L forskolin‐induced CRE response, in the absence (open circles) or presence (solid circles) of diazepam and: the calmodulin (CaM) inhibitor W‐7 hydrochloride, IP_3_ receptor antagonist 2‐APB, the L‐type calcium channel inhibitor (*R*)‐(+)‐Bay K 8644, and the Ca^2+^/CaM‐dependent protein kinase II inhibitor KN‐93. * indicates statistically significant difference from vehicle. (E) 0.1 μmol/L phenylephrine‐induced CRE response, or (F) 10 μmol/L forskolin‐induced CRE response, in the absence (open circles) or presence (solid circles) of diazepam and/or PDE inhibitors IBMX and rolipram. * Indicates statistically significant difference between the indicated groups. Points represent mean values from replicate experiments, horizontal lines the means and error bars the standard deviations. Statistical differences were determined using one‐way ANOVA and Sidak multiple comparisons test

Next, we sought to define the mechanism by which diazepam was modulating CRE activation by using inhibitors of various potential targets. Co‐addition of the GABA_A_R antagonist bicuculline had no effect on the positive modulation of phenylephrine or forskolin by diazepam (Figure 6A,B). Voltage‐gated calcium channels, through a CaM‐dependent mechanism, are known to play a role in α_1_‐AR‐induced cAMP production and diazepam is thought to bind to some Ca^2+^ channels.^23^ Cotreatment of cells with the broad spectrum Ca^2+^ channel inhibitor benidipine significantly reduced both the phenylephrine response and the diazepam modulation of the CRE response in α_1A_‐AR expressing cells but had no effect on the positive modulation of forskolin‐stimulated CRE response in the same cells (Figure 6A,B). Similarly, the CaM inhibitor W‐7 hydrochloride significantly reduced both the phenylephrine response and the diazepam modulation of the CRE response in α_1A_‐AR expressing cells, probably by blocking the same pathway as the calcium channel inhibitor but had no significant effect on forskolin stimulation (Figure 6C,D). These results implicate Ca^2+^ channels in α_1_?AR‐induced CRE activation, however are unlikely to be driving diazepam mediated positive modulation as diazepam modulation of forskolin‐induced CRE was unaffected by benidipine or W‐7 hydrochloride. Other inhibitors such as the InsP3R antagonist 2‐APB, the L‐type calcium channel inhibitor (*R*)‐(+)‐Bay K 8644, and the Ca^2+^/CaM‐dependent protein kinase II inhibitor KN‐93, had no significant effect on the ability of diazepam to positively modulate the phenylephrine‐ or forskolin‐stimulated CRE responses (Figure 6C,D).

Diazepam is an inhibitor of PDEs, especially PDE‐4,^24^ and thus should increase the levels of cAMP in cells during the CRE assay, potentially explaining our observations. In this case, we would expect co‐addition of the broad‐spectrum PDE inhibitor IBMX, or the PDE‐4 inhibitor rolipram, to have no additional modulatory effect on top of diazepam treatment in our CRE assays. Indeed, in α_1A_‐AR expressing COS‐7 cells, IBMX and rolipram positively modulated the CRE response of phenylephrine to a similar level as diazepam (Figure 6E); however, cotreatment using IBMX or rolipram with diazepam had no additional positive modulatory effect on the phenylephrine response (Figure 6E). Similarly, IBMX and rolipram positively modulated the CRE response of forskolin on these same cells, but no additive effect was observed for either IMBX plus diazepam or rolipram plus diazepam (Figure 6F). In fact, cotreatment of rolipram and diazepam significantly decreased the forskolin‐induced CRE response compared to rolipram alone (Figure 6F), possibly indicating competition between diazepam and rolipram at the same binding site on PDE‐4. These data strongly suggest that the positive modulation of the phenylephrine‐induced α_1_‐AR CRE response by diazepam, and other benzodiazepines, is caused through inhibition of PDEs.

**Figure 7:**
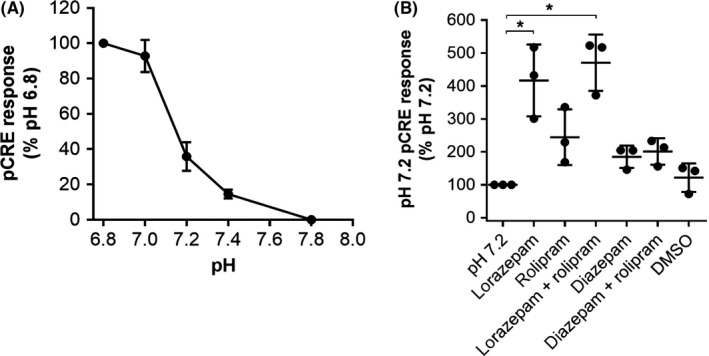
GPR68‐induced cAMP response element (CRE) response is modulated by lorazepam but not diazepam. (A) pH‐response curve of CRE stimulation in human embryonic kidney (HEK) 293T cells transfected with GPR68. Points are the mean ± SD of three independent experiments. (B) Modulation of pH 7.2 stimulation of the CRE response in HEK‐293T cells transfected with GPR68 and treated with lorazepam and diazepam in the presence or absence of rolipram. * Indicates statistically significant difference between the indicated groups as determined using one‐way ANOVA and Dunnett multiple comparisons test. Points represent mean values from individual replicate experiments, horizontal lines the mean of the replicates, and error bars the standard deviations

Lorazepam was recently reported to be an allosteric modulator of the pH‐sensitive GPCR, GPR68.^25^ GPR68 has been shown to couple to G_q_, G_s_, G_12/13,_ and G_i/o_ proteins^25^ and thus should activate the CRE reporter assay. We thus sought to use lorazepam modulation of GPR68 as a positive control for direct receptor allosterism in our CRE assay. A pH‐dependent CRE response was observed in HEK cells transfected with GPR68, with the observed EC_50_ of pH 7.2 reflective of other reports^25^ (Figure 7A). Critically lorazepam, but not diazepam, positively modulated the CRE response driven by pH 7.2 stimulation of GPR68 (Figure 7B). Treatment with both lorazepam and rolipram resulted in a CRE response similar to that of lorazepam alone. This contrasts with the competitive effect observed upon diazepam and rolipram cotreatment of phenylephrine‐stimulated α_1_‐AR expressing COS‐7 cells (Figure 6) and suggests that lorazepam modulates GPR68 through a PDE‐independent, possibly direct, mechanism.

## 4 DISCUSSION

The benzodiazepines diazepam, lorazepam and midazolam have been reported to be PAMs at the α_1_‐ARs.^7^ Waugh et al.^7^ used indirect radioligand binding and signaling assays on receptor‐overexpressing cell lines to demonstrate positive modulation of these benzodiazepines on α_1A_‐AR, α_1B_‐AR, and α_1D_‐AR; however, direct binding of the benzodiazepines to α_1_‐ARs has never been measured. Here, purified, thermostabilized α_1_‐AR variants were used as a tool to probe whether diazepam and lorazepam positively modulate α_1_‐AR signaling via direct binding to the receptors. Interestingly, no evidence was obtained that diazepam or lorazepam could bind to either purified α_1_‐AR subtype using fluorescent ligand binding assay (Figure 1) or STD NMR (Figure 2). Possibly, this may have been due to the thermostabilizing mutations in the receptors and/or the solubilization state in detergent micelles perturbing the natural benzodiazepine binding sites. In fact, agonists do exhibit weaker affinities for these thermostabilized receptors in solution^10^ and it is not clear whether the receptors can sample active states in detergent, which might preclude binding at allosteric sites. However, diazepam also had no effect on the binding of an orthosteric antagonist (QAPB) to cells stably expressing WT α_1B_‐AR and did not positively modulate phenylephrine‐induced intracellular Ca^2+^ release in cells expressing WT α_1A_‐AR or α_1B_‐AR (Figure 3), also suggesting that diazepam does not bind to these receptors.

Using a CRE reporter assay, which is responsive to multiple GPCR‐stimulated signaling pathways to detect downstream effects, diazepam was found to positively modulate phenylephrine‐induced CRE stimulation in WT α_1A_‐AR and α_1B_‐AR expressing cells. The ability of diazepam to modulate the CRE response was independent of α_1_‐AR stimulation, which was shown using β‐AR agonists and cells that natively express β‐ARs,^26^ and by directly activating AC with forskolin (Figure 4). This suggests that diazepam acts upon signaling elements downstream of the receptor. Interestingly, while diazepam treatment improved the potency of phenylephrine at activating CRE in both α_1A_‐AR and α_1B_‐AR expressing cells, it enhanced the efficacy of phenylephrine only at α_1B_‐AR expressing cells. This likely reflects differences between how phenylephrine activates CRE at α_1A_‐AR compared to α_1B_‐AR.

The SAR governing benzodiazepine action at GABA_A_ receptors is well understood. The substituent at C‐7 is of paramount importance; small electron‐withdrawing substituents at C‐7 generally impart high activity, whereas electron donors or large groups are inactive.^27^ Substitutions at N‐1 in contrast are generally tolerated, even though they can impart significant modulatory effects on GABA_A_ activity.^28^ By screening various analogues of diazepam, the SAR governing CRE modulation was found to be different to that at GABA_A_R (Table 1), with the methyl group at the N‐1 position of diazepam shown to be vital for modulating the CRE response, whereas phenyl substitution at C‐7 was tolerated. These chemical differences indicate that the effect observed here is through a target other than GABA_A_R.

Benzodiazepine interactions have been reported against cholecystokinin receptors,^29^ α_2_‐ARs,^30^ HIV‐1 reverse transcriptase,^30^ κ‐opioid receptors,^30^ muscarinic receptors,^30^ translocator protein,^31^ Ca^2+^ channels,^23^ Ca^2+^/CaM‐dependent protein kinases^32^, and PDE.^24^, ^33^ Using inhibitors of several of the other potential target proteins that could be responsible for CRE modulation we were able to conclude that inhibition of PDE, most likely PDE‐4, by diazepam causes modulation of the CRE response (Figure 6). Diazepam inhibits the activity of guinea pig PDE‐4 with an IC_50_ of 9 μmol/L,^24^ which is similar to the potency of diazepam for modulating the CRE response (Figure 4). Diazepam also competes with ^3^H‐rolipram at purified guinea pig PDE‐4,^24^ which potentially explains the ability of diazepam to compete with rolipram (Figure 6). Collado et al. screened several benzodiazepines for activity at guinea pig PDE‐4 and found that clonazepam, nitrazepam, and lorazepam, each of which are unsubstituted at N‐1, were less potent than diazepam,^24^ which broadly matches the SAR observed here. A similar benzodiazepine, lorazepam, also lacked the ability to modulate the CRE response, but was recently found to positively modulate the pH‐sensitive GPCR and GPR68. Here, we confirmed the activity of lorazepam upon GPR68 using the CRE assay and demonstrated that its activity is not due to PDE inhibition. Notably, lorazepam induced a significantly higher GPR68‐induced CRE response than diazepam or rolipram treatment. Interestingly, diazepam did not significantly increase the CRE response of GPR68 at pH 7.2, suggesting that GPR68 stimulates CRE in a different, more robust way to α_1_‐ARs. This may be related to the fact that GPR68 can couple to Gs proteins to directly activate cAMP accumulation, and thus CRE,^25^ whereas α_1_‐ARs stimulate CRE indirectly through Gq.

In summary, this work shows the value of stabilized, purified α_1_‐ARs to probe direct molecular interactions, allowing us to show that the modulation of α_1_‐AR activity by benzodiazepines in cell‐based assays is not a result of direct ligand binding. We further demonstrated that significant modulation of cellular CRE responses by diazepam most likely occurred through inhibition of PDE‐4. Given the widespread use of benzodiazepines therapeutically, this off‐target effect may contribute to their clinical actions and side effects and warrants further study. In contrast to this, lorazepam was validated as a direct‐acting, PAM of the pH sensitive receptor GPR68.
