# Supplementary material for: Diazepam is not a direct allosteric modulator of α1‐adrenoceptors, but modulates receptor signaling by inhibiting phosphodiesterase‐4
Source: Pharmacol Res Perspect. 2018 Dec 26;7(1):e00455. doi: 10.1002/prp2.455 (PMC6306559; doi:10.1002/prp2.455)
Supplement: Supplementary file 1 [file PRP2-7-e00455-s001.docx]

**Supporting information**

Benzodiazepine preparation

Lorazepam, oxazepam and temazepam were obtained from grinding tablets with a mortar and pestle, extraction with dichloromethane, evaporation of the solvent, and recrystallization from ethanol or ethanol/water.

Lorazepam: ^1^H NMR (400 MHz, CDCl_3_) δ 4.59 (1 H, d, *J* = 9.0 Hz, C-3), 5.06 (1 H, d, *J* = 9.0 Hz, O-H), 7.10-7.70 (7 H, m, Ar-H), 8.56 (1 H, s, N-H) ppm; ^13^C NMR (400 MHz, CDCl_3_) δ, 82.1, 122.7, 127.1, 128.9, 129.9, 130.2, 131.3, 131.4, 132.4, 133.2, 134.8, 137.4, 140.8, 164.2, 169.7 ppm.

Oxazepam: ^1^H NMR (600 MHz, CDCl_3_) δ 4.52 (1 H, d, J = 9.1 Hz, C-3), 5.02 (1 H, d, *J* = 9.1 Hz, O-H), 7.10-7.70 (8 H, m, Ar-H), 8.47 (1 H, s, N-H) ppm; ^13^C NMR (600 MHz, CDCl_3_) δ, 82.3, 123.0, 128.6, 129.2, 129.9, 130.1, 130.7, 131.2, 132.5, 135.6, 138.0, 164.4, 170.7 ppm.

Temazepam: ^1^H NMR (400 MHz, CDCl_3_) δ 3.48 (3 H, s, CH_3_), 4.74 (1 H, d, *J* = 9.1 Hz, C-3), 4.97 (1 H, d, *J* = 9.1 Hz, O-H), 7.30-7.65 (8 H, m, Ar-H) ppm; ^13^C NMR (400 MHz, CDCl_3_) δ, 35.3, 82.2, 122.9, 128.5, 129.6, 129.7, 130.2, 130.5, 131.0, 132.0, 137.3, 140.8, 163.5, 170.0 ppm.

7-Bromo-diazepam was synthesized in three steps. Treatment of 2-amino-5-bromobenzophenone with bromoacetyl bromide afforded a bromoacetamide; ammonolysis gave 7-bromo-nordiazepam; and finally methylation gave 7-bromo-diazepam.

2-(2-Bromoacetamido)-5-bromobenzophenone. Bromoacetyl bromide (0.0940 ml, 1.08 mmol) and 4 g of ice were added simultaneously and in portions to an ice-cold stirred solution of 2-amino-5-bromobenzophenone (200 mg, 0.72 mmol) at 0 ºC over 12 h. The organic layer was separated, washed with water, cold 1 M aq NaOH, dried (Na_2_SO_4_), and concentrated in vacuo. The crystalline residue was recrystallized from petroleum ether/chloroform (6 ml 1: 1) affording colorless needles of the title compound (243 mg, 85%). ^1^H NMR (400 MHz, CDCl_3_) δ 4.01 (2 H, s, CH_2_), 7.15-8.53 (8 H, m, Ar-H), 11.31 (1 H, s, N-H) ppm; ^13^C NMR (400 MHz, CDCl3) δ 29.3, 115.7, 123.3, 125.8, 128.6, 130.0, 133.2, 135.5, 136.7, 137.6, 138.4, 165.0, 197.8 ppm.

7-Bromo-1,3-dihydro-5-phenyl-2*H*-1,4-benzodiazepin-2-one. A solution of 2-(2-bromoacetamido)-5-bromobenzophenone (410 mg, 1.22 mmol) in diethyl ether (25 ml) was added to a solution of gaseous ammonia (1.78 g) dissolved in methanol (15 ml) at room temperature. After stirring for 20 h, the solution was concentrated in vacuo maintaining the temperature below 25 ºC. The residue was partitioned between diethyl ether and water, and the organic layer was separated, dried (Na_2_SO_4_), and concentrated in vacuo. The crude product was purified by flash chromatography (2: 2: 1 CH_2_Cl_2_: EtOAc: petroleum ether) affording the title compound (200 mg, 52%) as a yellow powder. ^1^H NMR (400 MHz, CDCl_3_) δ 4.32 (2 H, s, C-3), 7.10-7.65 (8 H, m, Ar-H), 9.43 (1 H, s, N-H) ppm; ^13^C NMR (400 MHz, CDCl_3_) δ 56.6, 116.3, 122.8, 128.4, 128.9, 129.6, 130.6, 133.7, 134.7, 137.8, 138.7, 169.7, 171.8 ppm.

7-Bromo-1,3-dihydro-1-methyl-5-phenyl-2*H*-1,4-benzodiazepin-2-one. A mixture of sodium hydroxide (60%, 9 mg, 0.220 mmol) and 7-bromo-1,3-dihydro-5-phenyl-2*H*-1,4-benzodiazepin-2-one (62 mg, 0.200 mmol) in dry dimethylformamide (2 mL) was kept at 0 ºC for 30 min under nitrogen atmosphere. The slurry was treated with methyl iodide (0.015 ml, 0.240 mmol) and stirred overnight at room temperature. Removal of the solvent under reduced pressure afford a brown gum that was partitioned between diethyl ether and saturated brine. The organic layer was separated, dried (MgSO_4_) and concentrated. Flash chromatography (2: 1: 1 CH_2_Cl_2_: EtOAc: petroleum ether) of the residue gave the title compound (55 mg, 84%) as a white powder. ^1^H NMR (400 MHz, CDCl_3_) δ 3.38 (3 H, s, CH_3_), 3.77 (1 H, d, *J* = 10.8 Hz, C-3), 4.83 (1 H, d, *J* = 10.8 Hz, C-3), 7.23-7.70 (8 H, m, Ar-H) ppm; ^13^C NMR (600 MHz, CDCl_3_) δ 35.0, 57.2, 121.2, 124.0, 128.4, 129.0, 129.8, 130.6, 130.7, 131.5, 139.1, 144.3, 170.4, 170.6 ppm.

7-Phenyl-diazepam, a GABA_AR_-inactive benzodiazepine, was synthesized from 4-phenylphenol. Benzoylation of 4-phenylphenol gave benzoyl 4-phenylbenzoate; Fries rearrangement gave 2-hydroxy-5-phenylbenzophenone; and a Smiles rearrangement of the 2-hydroxy-2-methylpropanamide ester employing the approach developed by Mizuno and Xie (Mizuno & Yamano, 2005) afforded 2-amino-5-phenylbenzophenone. This aniline was cyclized and methylated according to the approach of Sternbarch (Sternbach et al., 1962).

**Benzoyl 4-phenylbenzoate.** Benzoyl chloride (1.16 ml 10.0 mmol) was added in portions to a solution of 4-phenylphenol (1.70 g, 10.0 mmol) in diethyl ether (30 ml) and Et_3_N (3 ml). The mixture was stirred for 30 min at room temperature. Additional diethyl ether (50 ml) was added and the organic layer was washed with aq NaHCO_3_, separated and dried (MgSO_4_). The solvent was removed under reduced pressure and the residue was recrystallized from ethanol (20 ml) to give the title compound (2.64 g, 96%) as a white powder. ^1^H NMR (400 MHz, CDCl_3_) δ 7.28-8.25 (14 H, m, Ar-H) ppm; ^13^C NMR (400 MHz, CDCl_3_) δ 122.0, 127.1, 127.3, 128.2, 128.6, 128.8, 129.5, 130.2, 133.6, 139.0, 140.4, 150.4, 165.2 ppm.

**2-Hydroxy-5-phenylbenzophenone.** Benzoyl 4-phenylbenzoate (1.10 g, 4.00 mmol) was heated until melting at 180 ºC. AlCl_3_ (0.640 g, 4.80 mmol) was added and the temperature of the mixture was kept at 220 ºC for 16 h. The resultant black solid was ground into a powder, washed with cold 1 M HCl (aq) and extracted with dichloromethane (50 ml). The organic layer was separated, dried (MgSO_4_) and concentrated. Flash chromatography (1: 1 toluene: petroleum ether) of the residue afforded the title compound (593 mg, 54%) as a yellow powder. ^1^H NMR (400 MHz, CDCl_3_) δ 7.16-7.81 (13 H, m, Ar-H), 12.00 (1 H, s, OH) ppm; ^13^C NMR (400 MHz, CDCl_3_): δ 118.9, 119.2, 126.6, 127.1, 128.5, 128.9, 129.2, 131.7, 132.0, 132.1, 135.1, 137.8, 139.8, 162.6, 201.6 ppm.

**2-Amino-5-phenylbenzophenone.** A mixture of 2-hydroxy-5-phenylbenzophenone (1.00 g, 3.80 mmol) and Cs_2_CO_3_ (3.70 g, 11.4 mmol) in dry dimethylformamide (10 ml) was stirred for 30 min at room temperature. 2-Bromo-2-methylpropanamide (1.89 g, 11.4 mmol) was added and the mixture was heated at 90 ºC for 6 h. Additional NaOH (1.37 g, 34.2 mmol) was added in portions and the reaction mixture was kept at 140 ºC for 20 h. The solvents were removed under reduced pressure. The residue was extracted with diethyl ether and washed with cold 1 M HCl (aq). The organic layer was separated, dried (MgSO_4_) and concentrated. Flash chromatography (4: 4: 1 CH_2_Cl_2_: petroleum ether: EtOAc) of the residue gave the title compound (350 mg, 34%) as a yellow powder. ^1^H NMR (500 MHz, CDCl_3_) δ 6.12 (2 H, s, NH_2_), 6.84-7.75 (13 H, m, Ar-H) ppm; ^13^C NMR (500 MHz, CDCl_3_) δ 117.7, 118.4, 126.3, 126.6, 128.3, 128.7, 128.9, 129.4, 131.4, 132.7, 133.1, 140.0, 140.4, 150.3, 199.2 ppm.

**2-(2-Bromoacetamido)-5-phenylbenzophenone.** Bromoacetyl bromide (0.110 ml, 1.20 mmol) and 5 g of ice were added simultaneously and in portions to an ice-cold stirred solution of 2-amino-5-phenylbenzophenone (250 mg, 0.920 mmol) in diethyl ether (5 ml) at room temperature. The mixture was kept at 0 ºC for 12 h. The organic layer was separated, washed with water, cold 1 M aq NaOH, dried (Na_2_SO_4_), and concentrated in vacuo. The crystalline residue was recrystallized from petroleum ether and chloroform (6 ml 1: 1) to afford yellow needles of the title compound (296 mg, 82%). ^1^H NMR (600 MHz, CDCl_3_) δ 4.05 (2 H, s, CH_2_), 7.30-8.70 (13 H, m, Ar-H), 11.40 (1 H, s, N-H) ppm; ^13^C NMR (600 MHz, CDCl_3_) δ 29.4, 122.0, 124.6, 126.8, 127.7, 128.5, 129.0, 130.1, 131.7, 132.4, 132.8, 136.0, 138.2, 138.5, 139.2, 165.0, 199.1 ppm.

**7-Phenyl-1,3-dihydro-5-phenyl-2*H*-1,4-benzodiazepin-2-one.** A solution of 2-(2-bromoacetamido)-5-phenylbenzophenone (260 mg, 0.660 mmol) in diethyl ether (20 ml) was added to a solution of gaseous ammonia (1.10 g) dissolved in dry methanol (9 ml) at room temperature. After stirring for 20 h, the solution was concentrated in vacuo in a water bath below 25 ºC. The residue was partitioned between diethyl ether and water. The organic layer was separated, dried (MgSO_4_), and concentrated in vacuo. The crude product was purified by flash chromatography (2: 2: 1 CH_2_Cl_2_: EtOAc: petroleum ether) to afford the title compound (97 mg, 45%) as a white powder. ^1^H NMR (400 MHz, CDCl_3_) δ 4.39 (2 H, s, C-3), 7.25-7.74 (13 H, m, Ar-H), 9.53 (1 H, s, N-H) ppm; ^13^C NMR (400 MHz, CDCl_3_) δ 56.8, 121.7, 126.9, 127.5, 127.7, 128.3, 128.9, 129.6, 129.7, 130.4, 136.3, 138.0, 139.3, 139.4, 171.0, 172.1 ppm.

**7-Phenyl-1,3-dihydro-1-methyl-5-phenyl-2*H*-1,4-benzodiazepin-2-one.** 60% sodium hydroxide (7 mg, 0.165 mmol) was added to a solution of 7-phenyl-1,3-dihydro-5-phenyl-2*H*-1,4-benzodiazepin-2-one (50 mg, 0.15 mmol) in dry dimethylformamide (2 ml), and the mixture was kept at 0 ºC for 30 min under nitrogen atmosphere. The slurry was treated with methyl iodide (0.011 ml, 0.180 mmol) and stirred overnight at rt. Removal of the solvent under reduced pressure afforded a brown gum, which was partitioned between diethyl ether and saturated brine. The organic layer was separated, dried (MgSO_4_) and concentrated. Flash chromatography (2: 1: 1 CH_2_Cl_2_: EtOAc: petroleum ether) of the residue gave the title compound (44 mg, 86%) as a yellow powder. 1H NMR (400 MHz, CDCl_3_) δ 3.45 (3 H, s, CH3), 3.86 (1 H, d, *J* = 10.7 Hz, C-3), 4.85 (1 H, d, *J* = 10.7 Hz, C-3), 7.28-7.85 (13 H, m, Ar-H) ppm; ^13^C NMR (600 MHz, CDCl_3_) δ 34.8, 57.0, 121.4, 126.9, 127.8, 128.3, 128.6, 128.8, 129.1, 129.5, 129.9, 130.4, 136.7, 138.7, 139.1, 148.2, 170.1, 170.2 ppm.
